# Data that support the structural, chemical and morphological characterization and its influence on the electrochemical performance of stabilized Pd_x_Pt_1-x_ alloys as electrode materials for methanol oxidation in alkaline medium

**DOI:** 10.1016/j.dib.2020.105172

**Published:** 2020-01-25

**Authors:** J.J. De la Cruz-Cruz, M.A. Domínguez-Crespo, E. Ramírez-Meneses, A.M. Torres-Huerta, S.B. Brachetti-Sibaja, N. Cayetano-Castro, H.J. Dorantes-Rosales

**Affiliations:** a Instituto Politécnico Nacional, Centro de Investigación en Ciencia Aplicada y Tecnología Avanzada CICATA, Unidad Altamira, Carretera Tampico-Puerto Industrial, km 14.5, Altamira, Tamaulipas, CP 89600, Mexico; b Instituto Politécnico Nacional, UPIIH, Ciudad del Conocimiento y la Cultura, Carretera Pachuca-Actopan km, 1+500, San Agustin Tlaxiaca, Hidalgo, Mexico; c Departamento de Ingeniería Química, Industrial y de Alimentos, Universidad Iberoamericana, Prolongación Paseo de la Reforma 880, Lomas de Santa Fe, 01219, Ciudad de México, Mexico; d TecNM, Instituto Tecnológico de Cd. Madero. Ave. Primero de Mayo s/n Col. Los Mangos Cd., Madero Tamps, C.P 89440, Mexico; e Instituto Politécnico Nacional, Centro de Nanociencias Micro y Nanotecnologías, C.P 07300, Ciudad de México, Mexico; f Instituto Politécnico Nacional, ESIQIE, Departamento de Metalurgia, C.P 07300, Ciudad de México, Mexico

**Keywords:** Fuel cells, Methanol oxidation reaction, Alkaline media, Pt base catalysts, Organometallic method

## Abstract

Structural, compositional, morphological and electrochemical characterization are important to determinate the influence of platinum in the methanol oxidation in alkaline media. These data and analysis support the research article catalytic performance of alloyed Pt_x_Pd_1-x_ nanostructures supported on Vulcan XC-72R for the methanol oxidation in alkaline medium [1]. The data here presented included changes in the chemical composition, structure and microstructure. Also, complement data of cyclic voltammograms during activation in alkaline media as well as in presence of 1 M CH_3_OH to observe CO tolerance and Electrochemical Impedance Spectroscopy measurements at two different overpotentials (0.2 and 0.3 mV) on the onset potential for methanol electro-oxidation are published in this paper. The data can be used as a reference to determinate the effect of added different amounts of Pd to Pt/C catalysts, using an organometallic compounds method and octylamine as stabilizer. The data provided in this article have not been previously published and are available to enable critical or extended analyses.

**Table:** Specifications Table

Subject area	*Materials science, Nanostructures, electrochemistry*
More specific subject area	*Electrocatalyst, Direct methanol fuel cell, DMFC*
Type of data	***Tables, Figures and Text file***
How data were acquired	*Derived from a Mexican Government agreement through Laboratory Experiments. The characterization was realized by X-ray photoelectron spectroscopy (XPS, Microtech Multilab ESCA 2000), X-ray diffraction (XRD, Brucker D8 Advanced), Transmission electron microscopy (JEM-ARM2*00CF*, JEOL operating at* 200 kV*). Electrochemical measurements: cyclic voltammetry and Electrochemical Impedance Spectroscopy were performed in a pontentiostat/galvanostat (AUTOLAB Metrohm, 50,404).*
Data format	*Raw, filtered, fitted curves and analyzed data.*
Experimental factors	*The chemical and microstructural data were acquired on synthesized samples containing different amounts of Pd (30, 50 and 70 wt %) in Pt catalysts. The electrochemical data were obtained using 50*th *potential cycles for stabilization, and 50*th *cycles in presence of methanol* at scan rate of 10 mV s^−1^. The EIS measurements were acquired at two different over potentials (0.2 and 0.3 mV) on the onset potential for methanol electro-oxidation.
Experimental features	*The relationship between chemical, structural morphological and electrochemical performance of Pt*_*x*_*Pd*_*1-x*_*electrode materials The data were acquired in the as-prepared samples without special treatment.*
Data source location	*XPS spectra were taken at Centro de Nanociencias y Nanotecnología, Carr. Tijuana-Ensenada km 107, Playitas, 22,860, Ensenada B.C.* *TEM images were taken at the Centro de Nanociencias micro y Nanotecnologías del Instituto Politécnico Nacional C.P. 07300 México, DF, México.* *CV, EIS techniques and XRD patterns were collected at Centro de investigación en Ciencia Aplicada y Tecnología Avanzada del Instituto Politécnico Nacional, C.P. 89600 Altamira, Tamaulipas, México.*
Data accessibility	*All data are available with this article*
Related research article	J. J. De la Cruz-Cruz, M. A. Domínguez-Crespo, E. Ramírez-Meneses, A. M. Torres-Huerta, S. B. Brachetti Sibaja, N. Cayetano-Castro, H. Dorantes-Rosales. Efficient stabilization of in situ fabrication of Pt_x_Pd_1-x_ nanostructures for electro-oxidation of methanol in alkaline medium. International Journal of Hydrogen Energy, https://doi.org/10.1016/j.ijhydene.2019.12.087. In press.

**Table:** 

**Value of the Data** • The data are valuable because show changes in the crystallite size with different amounts of Pd to Pt/C catalysts when an organometallic approach is used in presence of octylamine as stabilizer. • The data also show differences in the nominal composition due to the formation of surface oxide compounds. • The data can be used to correlate microstructure with the electrochemical performance. • The data could be used to obtain new electrode materials for Methanol oxidation reaction in alkaline media.

## 1 Data

**Fig. 1:**
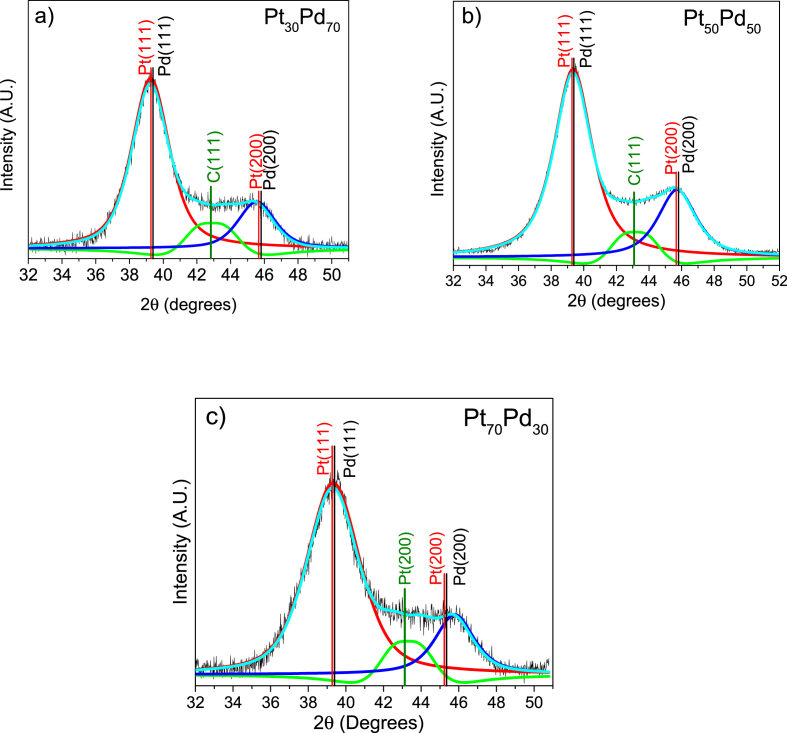
Deconvolution of X-ray diffraction patterns using the PseudoVoigt equation of a) Pt_30_Pd_70_, b) Pt_50_Pd_50_ and c) Pt_70_Pd_30_.

The data set of the deconvolution XRD to separate the signal of carbon from Pt_x_Pd_1-x_ bimetallic materials and used to determinate the crystallite size is shown in Fig. 1a–c. Small changes in the intensities and widening with the amount of Pd were observed, which provokes a reduction in the crystallite size.

**Fig. 2:**
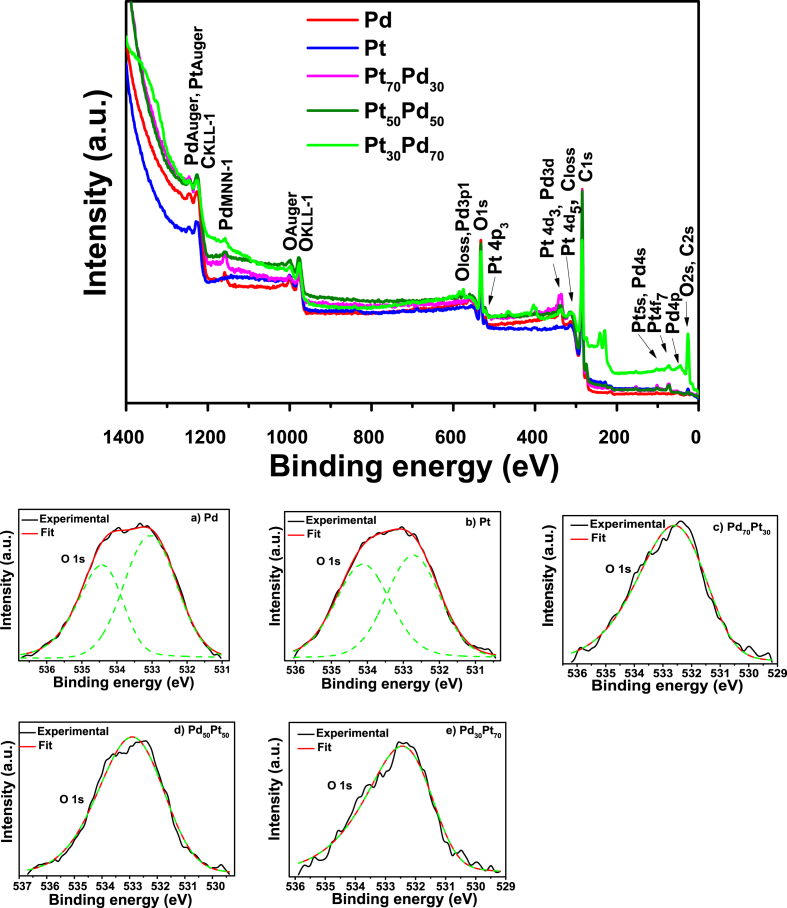
XPS Survey spectra of a) Pd, b) Pt, c) Pt_30_Pd_70_, d) Pt_50_Pd_50_ and e) Pt_70_Pd_30_ and deconvolution of O1s spectra.

Fig. 2 displays XPS survey spectra recorded for the surface of as-obtain mono- and bi-metallic materials. From these spectra, the range of binding energy for each metal composition in the high resolution was determinate and observed changes in the electronic properties during alloy formation [2].

**Fig. 3:**
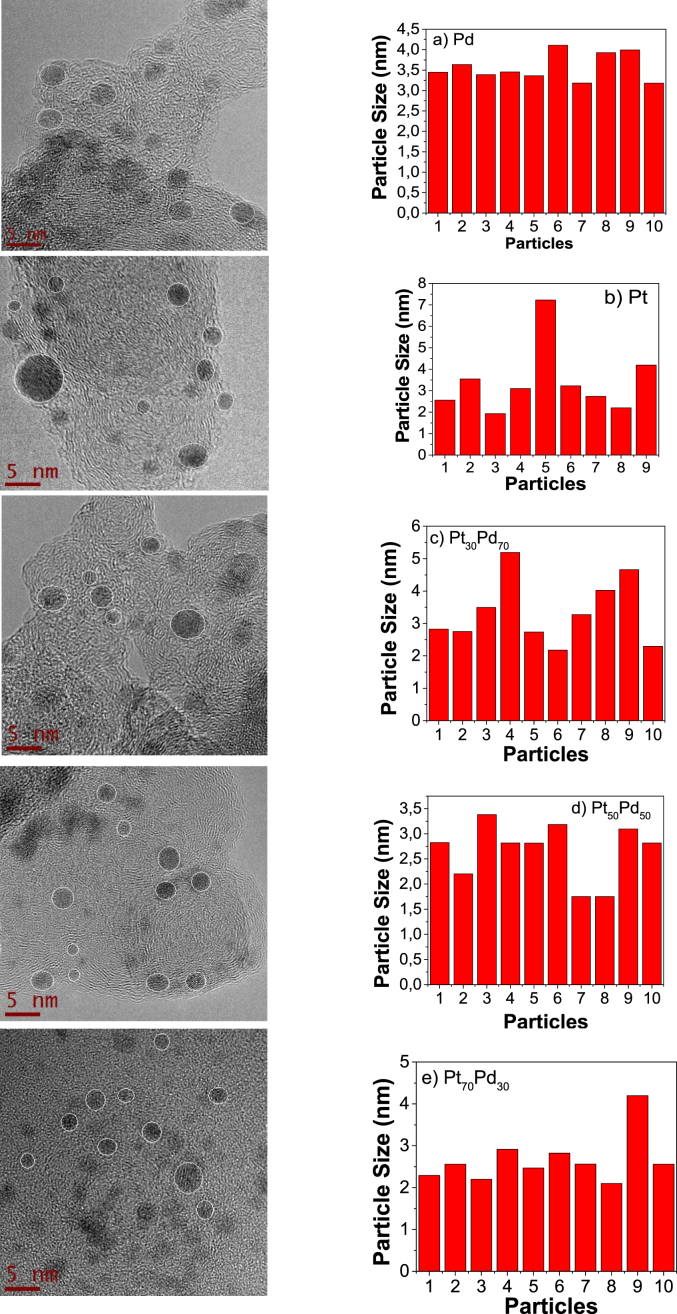
HRTEM morphologies of the mono and bimetallic nanocatalysts obtained from ligand displacement method and its corresponding average particle size.

Fig. 3 shows the semispherical morphology of the Pt_x_Pd_1-x_ nanostructures and the average particle size, using at least 10 particles [3].

**Fig. 4:**
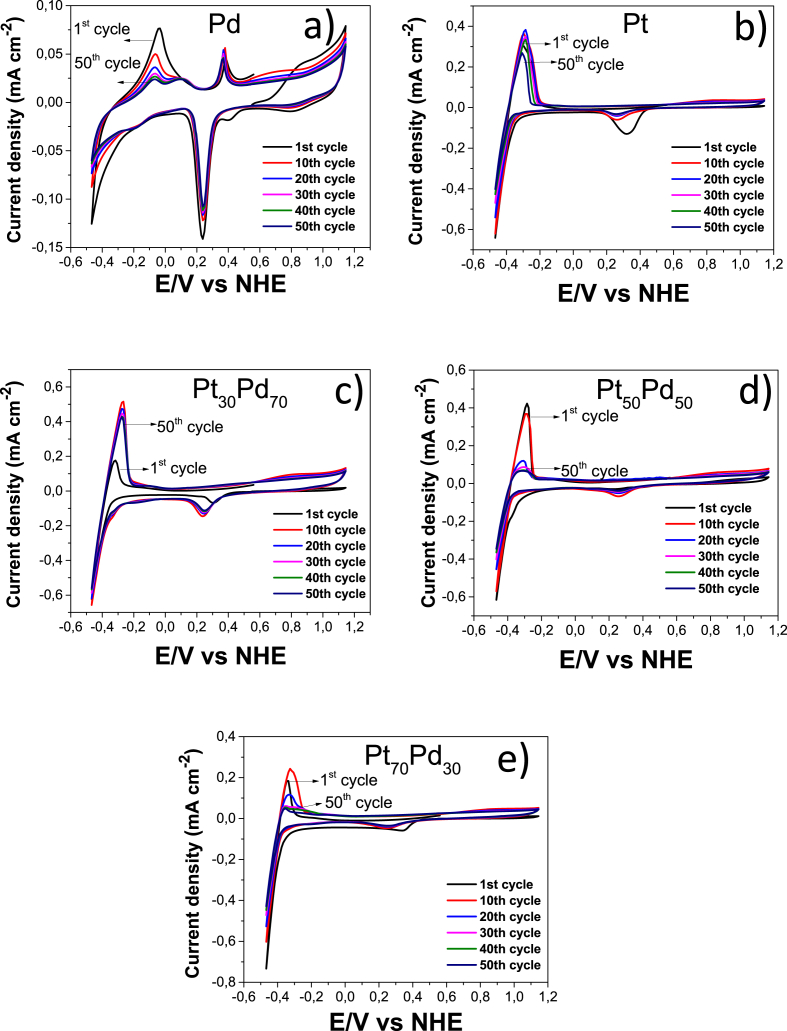
Cyclic voltammograms of a) Pd, b) Pt, c) Pt_30_Pd_70_, d) Pt_50_Pd_50_ and e) Pt_70_Pd_30_, evaluated in 1 M KOH at 25 °C using a scan rate of 10 mV s^−1^.

Fig. 4 shows the CV diagrams realized for the stabilization of the electrode materials in N_2_ purge KOH (1 M) electrolyte during 50th potential cycles. The synergistic effect by the Pd addition is only observed with a nominal composition of Pt_30_Pd_70,_ which it is also most stable than the other electrodes [4].

**Fig. 5:**
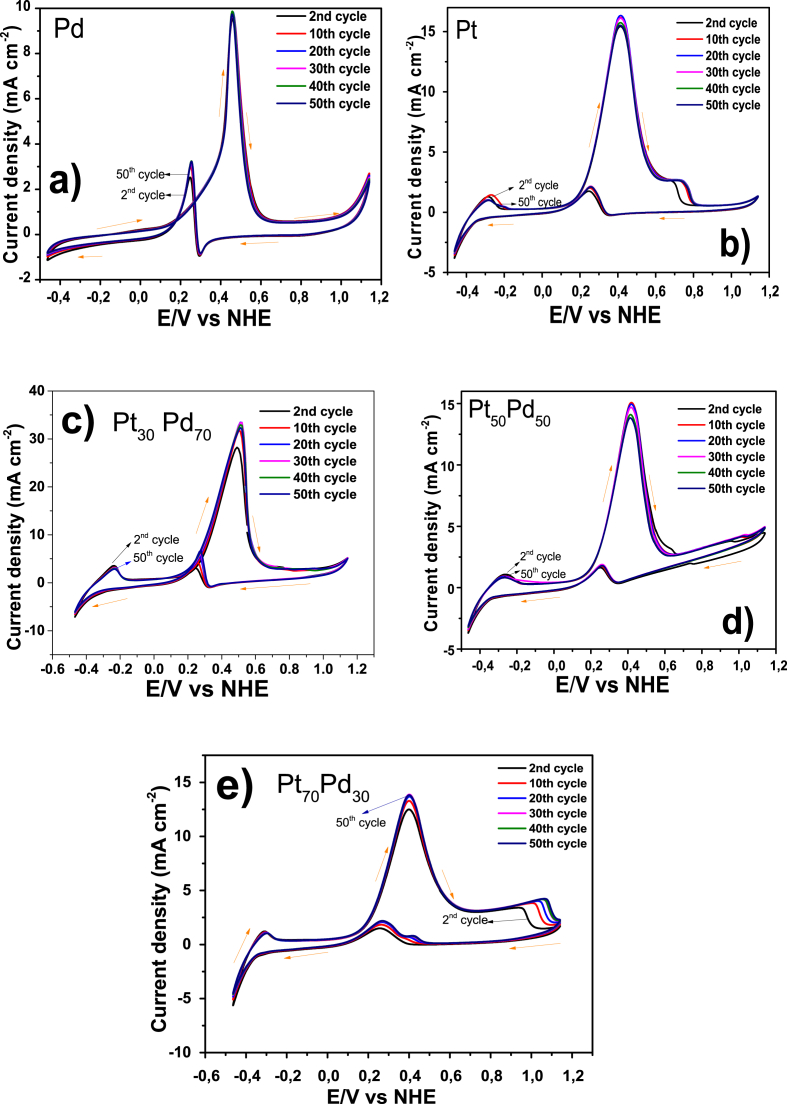
CV diagrams of the supported electrode materials a) Pd, b) Pt, c) Pt_30_Pd_70_, d) Pt_50_Pd_50_ and e) Pt_70_Pd_30_ in 1 M KOH + 1 M CH_3_OH at 25 °C using a scan rate of 10 mV s^−1^.

In Fig. 5, it is seen the electrocatalytic behavior of the mono- and bi-metallic materials on MOR and its evolution after 50th potential cycles [5].

## 2 Experimental design, materials and methods

### 2.1 Preparation of electrode materials

The catalysts were prepared by ligands displacement of organometallic compounds [6]. The metallic catalysts were prepared in-situ on Vulcan Carbon (XC 72R) which was adjusted to obtain a 10 wt% of metallic load and 90 wt% of support. The bimetallic precursors Pt_2_(dba)_3_ and Pd(dba)_2_ were synthesized from K_2_PtCl_4_ and Pd(dba)_2_. The precursors were mixed with THF anhydrous in a Fisher-Porter reactor, for starting the reaction, the reactor was filled with H_2_ at 3 bar, for 20 hours. Then, the solution was concentrated to separate the metallic powders, purified with anhydrous pentane and dried under reduced pressure. All the reagents were acquired from Sigma-Aldrich, Inc. and specific details of the experimental procedure were presented in reference [1].

### 2.2 Microstructure and chemical characterization

Powders were characterized by XRD (Bruker Advanced D8) with a Lynxeye detector and Cu Kα radiation (λ = 0.15406 nm) at a range of 20°–100° (2θ) at 40 kV and 40 kA and a scan rate of 0.021 min^−1^. The microstructure of the as-synthesized Pt_x_Pd_1-x_ powders was investigated by means of a JEM-ARM200CF, JEOL electron microscope, operating at 200 kV.

The chemical composition of the films was characterized by XPS using a commercial VG Microtech Multilab ESCA 2000 with a CLAM MCD detector, Al Kα radiation (1486.6 eV), operating at 1 × 10^−8^ Torr. Survey scans were obtained in the range of 0–1400 eV, with an energy step of 1.0 eV, and pass energy of 100 eV. Collected data were analyzed with a Shirley background subtraction, performed with a Gaussian-Lorentzian profile.

### 2.3 Electrochemical characterization

Electrochemical experiments were carried out in a standard three-electrode cell at room temperature. After preparation, the electrodes were rinsed, and their surface protected by a drop of water before being transferred through the air to the electrochemical cell. A platinum mesh was used as counter electrode and Hg/HgSO_4_ as reference electrode. All the working electrodes had a nominal mass of 0.1 mg cm^−2^.

The activation of electrodes was realized by cyclic voltammetry using an AUTOLAB (Metrohm, 50,404) pontentiostat/galvanostat in the potential range from −1.200 V to 700 V vs Hg/HgSO_4_ in 1.0 M KOH deareated solution in absence and presence of methanol (1.0 M) at scan rate of 10 mV s^−1^. To adequate comparison with the literature, all the potentials were converted to the scale of normal hydrogen electrode (NHE).
